# Analytic Computation of Vibrational Circular Dichroism Spectra Using Configuration Interaction Methods

**DOI:** 10.1021/acs.jpca.5c07287

**Published:** 2026-01-15

**Authors:** Brendan M. Shumberger, T. Daniel Crawford

**Affiliations:** Department of Chemistry, 1757 Virginia Tech, Blacksburg, Virginia 24061, United States

## Abstract

In this work, we present the first derivation and implementation of analytic-gradient methods for the computation of the atomic axial tensors (AATs) required for simulations of vibrational circular dichroism (VCD) spectra using configuration interaction methods including double (CID) and single and double (CISD) excitations. Our new implementation includes the use of noncanonical perturbed orbitals to improve the numerical stability of the gradients in the presence of orbital near-degeneracies, as well as frozen-core capabilities. We validated our analytic CID and CISD formulations against two new finite-difference approaches. Using this new implementation, we investigated the significance of singly excited determinants and the role of CI-coefficient optimization in VCD simulations by comparisons among the Hartree–Fock (HF) theory, second-order Møller–Plesset perturbation (MP2) theory, CID, and CISD theories. For our molecular test set including (*P*)-hydrogen peroxide, (*S*)-methyloxirane, (*R*)-3-chloro-1-butene, (*R*)-4-methyl-2-oxetanone, and (*M*)-1,3-dimethylallene, we noted sign discrepancies between the HF and MP2 methods compared with that of the new CID and CISD methods for four of the five molecules as well as similar discrepancies between the CID and CISD methods for (*M*)-1,3-dimethylallene.

## Graphical abstract

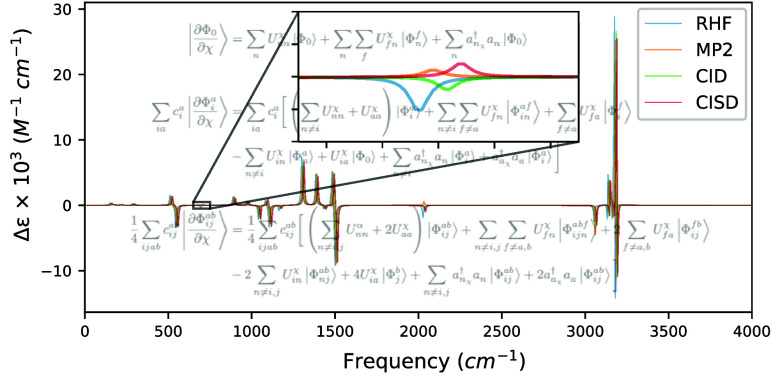

## 1 Introduction

Vibrational circular dichroism (VCD), the differential absorption of circularly polarized infrared radiation, is among the most widely used spectroscopic techniques for determining the absolute stereochemical configurations of chiral compounds.^1–7^ However, unlike other chiroptical spectroscopic methods such as optical rotation,^8–12^ electronic circular dichroism,^13–17^ and Raman optical activity,^18–21^ theoretical development of VCD simulations has, until recently, been limited to density functional theory (DFT) methods.

The principal challenge in implementing VCD within any quantum chemical approach, whether Hartree–Fock (HF), DFT, or more advanced wave function-based methods, is that the magnetic dipole transition moment unphysically vanishes within the Born–Oppenheimer approximation. To date, a number of general approaches for overcoming this complication have been developed;^22–26^ however, the most prominent solution to this problem was developed by Stephens in 1985, who proposed first-order perturbations with respect to magnetic fields and nuclear displacements be included in the ground-state wave function.^1^ By relating the first-order perturbations back to Taylor series expansions, the magnetic dipole transition moment could be formulated as an overlap between ground-state wave function derivatives with respect to nuclear coordinates and an external magnetic field, known as the atomic axial tensor (AAT). Shortly after Stephens’s breakthrough, the AAT was implemented using HF theory,^27–29^ multiconfigurational self-consistent field (MCSCF) theory,^30^ and density functional theory,^31^ bringing VCD to life in these methods. However, for some problems, higher accuracy was still desired, and methods which mixed calculations of the AAT, the atomic polar tensor (APT) (the second derivative of the energy with respect to nuclear coordinates and an external electric field), and vibrational Hessian were developed.^32^

Due to the complicated formulation of the AAT, almost 30 years passed before further implementation into wave function-based methods that include dynamical electron correlation, second-order Møller–Plesset perturbation theory (MP2), and configuration interaction including double excitations (CID) using a finite-difference approach.^33^ Due to the impractical computational scaling of numerical differentiation for wave functions involving determinantal expansions [*O*(*N*)^11^], Shumberger, Pearce, and Crawford developed an analytic formulation based on derivatives of excited determinants using a second-quantized approach, which they validated through the finite-difference formulation reducing the computational scaling to *O*(*N*)^5^ (given the appropriate intermediates).^34^

The purpose of the present work is to extend these methods for the first time to the configuration interaction singles and doubles (CISD) method in order to examine the significance of singly excited determinants for VCD rotatory strengths. We note that any of the general approaches to circumventing the vanishing magnetic dipole transition moment mentioned above may be implemented at the CI level of theory, and we have chosen to begin with Stephens’s formulation. In addition, we report additional improvements upon previous implementations, allowing the use of noncanonical perturbed orbitals,^35^ which provide greater numerical stability in the case of near-degenerate orbitals, and frozen-core for greater computational efficiency and for use with basis sets designed for the treatment of valence correlation (e.g., the cc-pVXZ basis sets of Dunning and co-workers^36^).

## 2 Theory

From Stephens’s formulation of VCD, the magnetic dipole transition moment includes the quantity *I*_αβ_^λ^, which is the electronic contribution to the atomic axial tensor (AAT). This quantity is formulated as the overlap between ground-state wave function derivatives where the bra-state wave function is differentiated with respect to nuclear displacements, *R*_λα_, where α is a Cartesian coordinate of the λ-th nucleus, and the ket-state wave function is differentiated with respect to the β-th Cartesian direction of the magnetic field, *H*_β_,

1 $$I_{\alpha \beta }^{\lambda }=\left\langle {\left(\frac{\partial \Psi_{G}(\mathrm{R⃗})}{\partial R_{\lambda \alpha }}\right)}_{R_{\lambda \alpha }=R_{\lambda \alpha }^{0}}|(\frac{\partial \Psi_{G}\left({\mathrm{R⃗}}_{0},H_{\beta }\right)}{\partial H_{\beta }}{)}_{H_{\beta }=0}\right\rangle$$

By approximating the wave function, Ψ_*G*_, as an intermediately normalized CISD wave function,

2 $$\left|\Psi_{G}\left(\mathrm{R⃗},H_{\beta }\right)\right\rangle \approx \left|\Phi_{0}\right\rangle +{Ĉ}_{1}\left|\Phi_{0}\right\rangle +{Ĉ}_{2}\left|\Phi_{0}\right\rangle$$

we can obtain the CISD AAT where the *Ĉ*_1_ and *Ĉ*_2_ operators are

3 $${Ĉ}_{1}=\sum_{\mathrm{ia}}c_{i}^{a}a_{a}^{†}a_{i}$$

and

4 $${Ĉ}_{2}=\frac{1}{4}\sum_{\mathrm{ijab}}c_{\mathrm{ij}}^{\mathrm{ab}}a_{a}^{†}a_{b}^{†}a_{j}a_{i}$$

and *i*, *j*, ... (*a*, *b*, ...) denote occupied (unoccupied) spin orbitals. Insertion of eq 2 into eq 1 and evaluation of the operators lead to an expression for the AAT in terms of CI coefficients, derivatives of CI coefficients, determinants, and derivatives of determinants, i.e.,

5 $$\begin{array}{l} {I_{\alpha \beta }^{\lambda }}_{\mathrm{int}}=\langle \frac{\partial \Phi_{0}}{\partial R_{\lambda \alpha }}\left|\frac{\partial \Phi_{0}}{\partial H_{\beta }}\right\rangle +\sum_{\mathrm{ia}}\frac{\partial {c_{i}^{a}}^{†}}{\partial R_{\lambda \alpha }}\langle \Phi_{i}^{a}\left|\frac{\partial \Phi_{0}}{\partial H_{\beta }}\right\rangle +\sum_{\mathrm{ia}}{c_{i}^{a}}^{†}\langle \frac{\partial \Phi_{i}^{a}}{\partial R_{\lambda \alpha }}\left|\frac{\partial \Phi_{0}}{\partial H_{\beta }}\right\rangle +\sum_{\mathrm{ia}}\frac{\partial c_{i}^{a}}{\partial H_{\beta }}\left\langle \frac{\partial \Phi_{0}}{\partial R_{\lambda \alpha }}|\Phi_{i}^{a}\right\rangle +\sum_{\mathrm{ia}}c_{i}^{a}\langle \frac{\partial \Phi_{0}}{\partial R_{\lambda \alpha }}\left|\frac{\partial \Phi_{i}^{a}}{\partial H_{\beta }}\right\rangle \\ +\frac{1}{4}\sum_{\mathrm{ijab}}\frac{\partial {c_{\mathrm{ij}}^{\mathrm{ab}}}^{†}}{\partial R_{\lambda \alpha }}\langle \Phi_{\mathrm{ij}}^{\mathrm{ab}}\left|\frac{\partial \Phi_{0}}{\partial H_{\beta }}\right\rangle +\frac{1}{4}\sum_{\mathrm{ijab}}{c_{\mathrm{ij}}^{\mathrm{ab}}}^{†}\langle \frac{\partial \Phi_{\mathrm{ij}}^{\mathrm{ab}}}{\partial R_{\lambda \alpha }}\left|\frac{\partial \Phi_{0}}{\partial H_{\beta }}\right\rangle +\frac{1}{4}\sum_{\mathrm{ijab}}\frac{\partial c_{\mathrm{ij}}^{\mathrm{ab}}}{\partial H_{\beta }}\left\langle \frac{\partial \Phi_{0}}{\partial R_{\lambda \alpha }}|\Phi_{\mathrm{ij}}^{\mathrm{ab}}\right\rangle +\frac{1}{4}\sum_{\mathrm{ijab}}c_{\mathrm{ij}}^{\mathrm{ab}}\langle \frac{\partial \Phi_{0}}{\partial R_{\lambda \alpha }}\left|\frac{\partial \Phi_{\mathrm{ij}}^{\mathrm{ab}}}{\partial H_{\beta }}\right\rangle \\ +\sum_{\mathrm{ia}}\sum_{\mathrm{kc}}\frac{\partial {c_{i}^{a}}^{†}}{\partial R_{\lambda \alpha }}\frac{\partial c_{k}^{c}}{\partial H_{\beta }}\left\langle \Phi_{i}^{a}|\Phi_{k}^{c}\right\rangle +\sum_{\mathrm{ia}}\sum_{\mathrm{kc}}\frac{\partial {c_{i}^{a}}^{†}}{\partial R_{\lambda \alpha }}c_{k}^{c}\langle \Phi_{i}^{a}\left|\frac{\partial \Phi_{k}^{c}}{\partial H_{\beta }}\right\rangle +\sum_{\mathrm{ia}}\sum_{\mathrm{kc}}{c_{i}^{a}}^{†}\frac{\partial c_{k}^{c}}{\partial H_{\beta }}\left\langle \frac{\partial \Phi_{i}^{a}}{\partial R_{\lambda \alpha }}|\Phi_{k}^{c}\right\rangle +\sum_{\mathrm{ia}}\sum_{\mathrm{kc}}{c_{i}^{a}}^{†}c_{k}^{c}\langle \frac{\partial \Phi_{i}^{a}}{\partial R_{\lambda \alpha }}\left|\frac{\partial \Phi_{k}^{c}}{\partial H_{\beta }}\right\rangle \\ +\frac{1}{4}\sum_{\mathrm{ijab}}\sum_{\mathrm{kc}}\frac{\partial {c_{\mathrm{ij}}^{\mathrm{ab}}}^{†}}{\partial R_{\lambda \alpha }}\frac{\partial c_{k}^{c}}{\partial H_{\beta }}\left\langle \Phi_{\mathrm{ij}}^{\mathrm{ab}}|\Phi_{k}^{c}\right\rangle +\frac{1}{4}\sum_{\mathrm{ijab}}\sum_{\mathrm{kc}}\frac{\partial {c_{\mathrm{ij}}^{\mathrm{ab}}}^{†}}{\partial R_{\lambda \alpha }}c_{k}^{c}\langle \Phi_{\mathrm{ij}}^{\mathrm{ab}}\left|\frac{\partial \Phi_{k}^{c}}{\partial H_{\beta }}\right\rangle +\frac{1}{4}\sum_{\mathrm{ijab}}\sum_{\mathrm{kc}}{c_{\mathrm{ij}}^{\mathrm{ab}}}^{†}\frac{\partial c_{k}^{c}}{\partial H_{\beta }}\left\langle \frac{\partial \Phi_{\mathrm{ij}}^{\mathrm{ab}}}{\partial R_{\lambda \alpha }}|\Phi_{k}^{c}\right\rangle \\ +\frac{1}{4}\sum_{\mathrm{ijab}}\sum_{\mathrm{kc}}{c_{\mathrm{ij}}^{\mathrm{ab}}}^{†}c_{k}^{c}\langle \frac{\partial \Phi_{\mathrm{ij}}^{\mathrm{ab}}}{\partial R_{\lambda \alpha }}\left|\frac{\partial \Phi_{k}^{c}}{\partial H_{\beta }}\right\rangle +\frac{1}{4}\sum_{\mathrm{ia}}\sum_{\mathrm{klcd}}\frac{\partial {c_{i}^{a}}^{†}}{\partial R_{\lambda \alpha }}\frac{\partial c_{\mathrm{kl}}^{\mathrm{cd}}}{\partial H_{\beta }}\left\langle \Phi_{i}^{a}|\Phi_{\mathrm{kl}}^{\mathrm{cd}}\right\rangle +\frac{1}{4}\sum_{\mathrm{ia}}\sum_{\mathrm{klcd}}\frac{\partial {c_{i}^{a}}^{†}}{\partial R_{\lambda \alpha }}c_{\mathrm{kl}}^{\mathrm{cd}}\langle \Phi_{i}^{a}\left|\frac{\partial \Phi_{\mathrm{kl}}^{\mathrm{cd}}}{\partial H_{\beta }}\right\rangle \\ +\frac{1}{4}\sum_{\mathrm{ia}}\sum_{\mathrm{klcd}}{c_{i}^{a}}^{†}\frac{\partial c_{\mathrm{kl}}^{\mathrm{cd}}}{\partial H_{\beta }}\left\langle \frac{\partial \Phi_{i}^{a}}{\partial R_{\lambda \alpha }}|\Phi_{\mathrm{kl}}^{\mathrm{cd}}\right\rangle +\frac{1}{4}\sum_{\mathrm{ia}}\sum_{\mathrm{klcd}}{c_{i}^{a}}^{†}c_{\mathrm{kl}}^{\mathrm{cd}}\langle \frac{\partial \Phi_{i}^{a}}{\partial R_{\lambda \alpha }}\left|\frac{\partial \Phi_{\mathrm{kl}}^{\mathrm{cd}}}{\partial H_{\beta }}\right\rangle +\frac{1}{16}\sum_{\mathrm{ijab}}\sum_{\mathrm{klcd}}\frac{\partial {c_{\mathrm{ij}}^{\mathrm{ab}}}^{†}}{\partial R_{\lambda \alpha }}\frac{\partial c_{\mathrm{kl}}^{\mathrm{cd}}}{\partial H_{\beta }}\left\langle \Phi_{\mathrm{ij}}^{\mathrm{ab}}|\Phi_{\mathrm{kl}}^{\mathrm{cd}}\right\rangle +\frac{1}{16}\sum_{\mathrm{ijab}}\sum_{\mathrm{klcd}}\frac{\partial {c_{\mathrm{ij}}^{\mathrm{ab}}}^{†}}{\partial R_{\lambda \alpha }}c_{\mathrm{kl}}^{\mathrm{cd}}\langle \Phi_{\mathrm{ij}}^{\mathrm{ab}}\left|\frac{\partial \Phi_{\mathrm{kl}}^{\mathrm{cd}}}{\partial H_{\beta }}\right\rangle +\frac{1}{16}\sum_{\mathrm{ijab}}\sum_{\mathrm{klcd}}{c_{\mathrm{ij}}^{\mathrm{ab}}}^{†}\frac{\partial c_{\mathrm{kl}}^{\mathrm{cd}}}{\partial H_{\beta }}\left\langle \frac{\partial \Phi_{\mathrm{ij}}^{\mathrm{ab}}}{\partial R_{\lambda \alpha }}|\Phi_{\mathrm{kl}}^{\mathrm{cd}}\right\rangle +\frac{1}{16}\sum_{\mathrm{ijab}}\sum_{\mathrm{klcd}}{c_{\mathrm{ij}}^{\mathrm{ab}}}^{†}c_{\mathrm{kl}}^{\mathrm{cd}}\langle \frac{\partial \Phi_{\mathrm{ij}}^{\mathrm{ab}}}{\partial R_{\lambda \alpha }}\left|\frac{\partial \Phi_{\mathrm{kl}}^{\mathrm{cd}}}{\partial H_{\beta }}\right\rangle \end{array}$$

Using the orbital derivative approach developed for the MP2 AAT^34^ and noting χ = *R*_*λα*_ or *H*_β_, we have that

6 $$\left|\frac{\partial \Phi_{0}}{\partial \chi }\right\rangle =\sum_{n}U_{\mathrm{nn}}^{\chi }\left|\Phi_{0}\right\rangle +\sum_{n}\sum_{f}U_{\mathrm{fn}}^{\chi }\left|\Phi_{n}^{f}\right\rangle +\sum_{n}a_{n_{\chi }}^{†}a_{n}\left|\Phi_{0}\right\rangle$$

In eq 6, operators of the form *a*_*p*_χ__^†^ are second-quantized operators that create orbitals of the form ϕ_*p*_^χ^, which denotes derivatives of the atomic-orbital basis functions transformed into the spin–orbital basis. By a similar procedure, the derivatives of singly excited and doubly excited determinants (with their corresponding CI coefficients) are

7 $$\sum_{\mathrm{ia}}c_{i}^{a}\left|\frac{\partial \Phi_{i}^{a}}{\partial \chi }\right\rangle =\sum_{\mathrm{ia}}c_{i}^{a}\left[\left(\sum_{n\neq i}U_{\mathrm{nn}}^{\chi }+U_{\mathrm{aa}}^{\chi }\right)\left|\Phi_{i}^{a}\right\rangle +\sum_{n\neq i}\sum_{f\neq a}U_{\mathrm{fn}}^{\chi }\left|\Phi_{\mathrm{in}}^{\mathrm{af}}\right\rangle +\sum_{f\neq a}U_{\mathrm{fa}}^{\chi }\left|\Phi_{i}^{f}\right\rangle -\sum_{n\neq i}U_{\mathrm{in}}^{\chi }\left|\Phi_{n}^{a}\right\rangle +U_{\mathrm{ia}}^{\chi }\left|\Phi_{0}\right\rangle +\sum_{n\neq i}a_{n_{\chi }}^{†}a_{n}\left|\Phi_{i}^{a}\right\rangle +a_{a_{\chi }}^{†}a_{a}\left|\Phi_{i}^{a}\right\rangle \right]$$

and

8 $$\frac{1}{4}\sum_{\mathrm{ijab}}c_{\mathrm{ij}}^{\mathrm{ab}}\left|\frac{\partial \Phi_{\mathrm{ij}}^{\mathrm{ab}}}{\partial \chi }\right\rangle =\frac{1}{4}\sum_{\mathrm{ijab}}c_{\mathrm{ij}}^{\mathrm{ab}}\left[\left(\sum_{n\neq i,j}U_{\mathrm{nn}}^{\alpha }+2U_{\mathrm{aa}}^{\chi }\right)\left|\Phi_{\mathrm{ij}}^{\mathrm{ab}}\right\rangle +\sum_{n\neq i,j}\sum_{f\neq a,b}U_{\mathrm{fn}}^{\chi }\left|\Phi_{\mathrm{ijn}}^{\mathrm{abf}}\right\rangle +2\sum_{f\neq a,b}U_{\mathrm{fa}}^{\chi }\left|\Phi_{\mathrm{ij}}^{\mathrm{fb}}\right\rangle -2\sum_{n\neq i,j}U_{\mathrm{in}}^{\chi }\left|\Phi_{\mathrm{nj}}^{\mathrm{ab}}\right\rangle +4U_{\mathrm{ia}}^{\chi }\left|\Phi_{j}^{b}\right\rangle +\sum_{n\neq i,j}a_{n_{\chi }}^{†}a_{n}\left|\Phi_{\mathrm{ij}}^{\mathrm{ab}}\right\rangle +2a_{a_{\chi }}^{†}a_{a}\left|\Phi_{\mathrm{ij}}^{\mathrm{ab}}\right\rangle \right]$$

respectively. We note that when χ = *H*_β_, terms involving the second-quantized core derivative operators will be zero since our atomic-orbital basis functions carry no dependence on the magnetic field, i.e., we have not used gauge-including atomic orbitals (GIAOs) at this stage. Inserting eqs 6–8 into eq 5 with the relevant perturbations and evaluating the contractions with both the standard Wick’s theorem contractions,^37^ in addition to those derived previously,^34^ allow for an expression for the intermediately normalized AAT to be derived.

9 $$\begin{array}{l} {I_{\alpha \beta }^{\lambda }}_{\mathrm{int}}=\sum_{m}\sum_{e}U_{\mathrm{em}}^{H_{\beta }}\left(U_{\mathrm{em}}^{R_{\lambda \alpha }}+\left\langle \phi_{m}^{R_{\lambda \alpha }}|\phi_{e}\right\rangle \right)+\sum_{\mathrm{ia}}\frac{\partial {c_{i}^{a}}^{†}}{\partial R_{\lambda \alpha }}U_{\mathrm{ai}}^{H_{\beta }}-\frac{1}{2}\sum_{\mathrm{ia}}{c_{i}^{a}}^{†}\left[\sum_{e}U_{\mathrm{ei}}^{H_{\beta }}\left(S_{\mathrm{ea}}^{R_{\lambda \alpha }}+\left\langle \phi_{a}^{R_{\lambda \alpha }}|\phi_{e}\right\rangle \right)-\sum_{m}U_{\mathrm{am}}^{H_{\beta }}\left(S_{\mathrm{im}}^{R_{\lambda \alpha }}+\left\langle \phi_{m}^{R_{\lambda \alpha }}|\phi_{i}\right\rangle \right)\right]\\ +\sum_{\mathrm{ia}}\frac{\partial c_{i}^{a}}{\partial H_{\beta }}\left(U_{\mathrm{ai}}^{R_{\lambda \alpha }}+\left\langle \phi_{i}^{R_{\lambda \alpha }}|\phi_{a}\right\rangle \right)+\sum_{\mathrm{ia}}\frac{\partial {c_{i}^{a}}^{†}}{\partial R_{\lambda \alpha }}\frac{\partial c_{i}^{a}}{\partial H_{\beta }}-\frac{1}{2}\sum_{\mathrm{ia}}\frac{\partial c_{i}^{a}}{\partial H_{\beta }}\left[\sum_{e}\left(S_{\mathrm{ae}}^{R_{\lambda \alpha }}+\left\langle \phi_{e}^{R_{\lambda \alpha }}|\phi_{a}\right\rangle \right){c_{i}^{e}}^{†}-\sum_{m}\left(S_{\mathrm{mi}}^{R_{\lambda \alpha }}+\left\langle \phi_{i}^{R_{\lambda \alpha }}|\phi_{m}\right\rangle \right){c_{m}^{a}}^{†}\right]\\ +\sum_{\mathrm{ia}}c_{i}^{a}\left[U_{\mathrm{ia}}^{H_{\beta }}\left[\sum_{\mathrm{kc}}\left(U_{\mathrm{kc}}^{R_{\lambda \alpha }}+\left\langle \phi_{c}^{R_{\lambda \alpha }}|\phi_{k}\right\rangle \right){c_{k}^{c}}^{†}\right]+\sum_{\mathrm{me}}U_{\mathrm{em}}^{H_{\beta }}[\left(U_{\mathrm{em}}^{R_{\lambda \alpha }}+\left\langle \phi_{m}^{R_{\lambda \alpha }}|\phi_{e}\right\rangle \right){c_{i}^{a}}^{†}-\left(U_{\mathrm{ei}}^{R_{\lambda \alpha }}+\left\langle \phi_{i}^{R_{\lambda \alpha }}|\phi_{e}\right\rangle \right){c_{m}^{a}}^{†}-\left(U_{\mathrm{am}}^{R_{\lambda \alpha }}+\left\langle \phi_{m}^{R_{\lambda \alpha }}|\phi_{a}\right\rangle \right){c_{i}^{e}}^{†}+\left(U_{\mathrm{ai}}^{R_{\lambda \alpha }}+\left\langle \phi_{i}^{R_{\lambda \alpha }}|\phi_{a}\right\rangle \right){c_{m}^{e}}^{†}]\right]\\ +\sum_{\mathrm{ijab}}\frac{\partial {c_{\mathrm{ij}}^{\mathrm{ab}}}^{†}}{\partial R_{\lambda \alpha }}U_{\mathrm{bj}}^{H_{\beta }}c_{i}^{a}+\sum_{\mathrm{ia}}\frac{\partial c_{i}^{a}}{\partial H_{\beta }}\left[\sum_{\mathrm{kc}}\left(U_{\mathrm{kc}}^{R_{\lambda \alpha }}+\left\langle \phi_{c}^{R_{\lambda \alpha }}|\phi_{k}\right\rangle \right)c_{\mathrm{ki}}^{\mathrm{ca}}\right]-\frac{1}{2}\sum_{\mathrm{ia}}c_{i}^{a}\left[-\sum_{\mathrm{kme}}U_{\mathrm{em}}^{H_{\beta }}\left(S_{\mathrm{km}}^{R_{\lambda \alpha }}+\left\langle \phi_{m}^{R_{\lambda \alpha }}|\phi_{k}\right\rangle \right){c_{\mathrm{ki}}^{\mathrm{ea}}}^{†}+\sum_{\mathrm{cme}}U_{\mathrm{em}}^{H_{\beta }}\left(S_{\mathrm{ec}}^{R_{\lambda \alpha }}+\left\langle \phi_{c}^{R_{\lambda \alpha }}|\phi_{e}\right\rangle \right){c_{\mathrm{im}}^{\mathrm{ac}}}^{†}\right]\\ +\sum_{\mathrm{ia}}\frac{\partial {c_{i}^{a}}^{†}}{\partial R_{\lambda \alpha }}\sum_{\mathrm{kc}}U_{\mathrm{kc}}^{H_{\beta }}c_{\mathrm{ki}}^{\mathrm{ca}}+\sum_{\mathrm{ijab}}\frac{\partial c_{\mathrm{ij}}^{\mathrm{ab}}}{\partial H_{\beta }}\left(U_{\mathrm{bj}}^{R_{\lambda \alpha }}+\left\langle \phi_{j}^{R_{\lambda \alpha }}|\phi_{b}\right\rangle \right){c_{i}^{a}}^{†}+\frac{1}{4}\sum_{\mathrm{ijab}}\frac{\partial {c_{\mathrm{ij}}^{\mathrm{ab}}}^{†}}{\partial R_{\lambda \alpha }}\frac{\partial c_{\mathrm{ij}}^{\mathrm{ab}}}{\partial H_{\beta }}-\frac{1}{4}\sum_{\mathrm{ijab}}\frac{\partial c_{\mathrm{ij}}^{\mathrm{ab}}}{\partial H_{\beta }}\left[-\sum_{k}\left(S_{\mathrm{ki}}^{R_{\lambda \alpha }}+\left\langle \phi_{i}^{R_{\lambda \alpha }}|\phi_{k}\right\rangle \right){c_{\mathrm{kj}}^{\mathrm{ab}}}^{†}+\sum_{c}\left(S_{\mathrm{ac}}^{R_{\lambda \alpha }}+\left\langle \phi_{c}^{R_{\lambda \alpha }}|\phi_{a}\right\rangle \right){c_{\mathrm{ij}}^{\mathrm{cb}}}^{†}\right]\\ +\frac{1}{2}\sum_{\mathrm{ijab}}{c_{\mathrm{ij}}^{\mathrm{ab}}}^{†}\frac{1}{2}\left[\sum_{\mathrm{me}}U_{\mathrm{em}}^{H_{\beta }}\left(U_{\mathrm{em}}^{R_{\lambda \alpha }}+\left\langle \phi_{m}^{R_{\lambda \alpha }}|\phi_{e}\right\rangle \right)c_{\mathrm{ij}}^{\mathrm{ab}}-\sum_{\mathrm{me}}U_{\mathrm{ej}}^{H_{\beta }}\left(U_{\mathrm{em}}^{R_{\lambda \alpha }}+\left\langle \phi_{m}^{R_{\lambda \alpha }}|\phi_{e}\right\rangle \right)c_{\mathrm{im}}^{\mathrm{ab}}-\sum_{\mathrm{me}}U_{\mathrm{bm}}^{H_{\beta }}\left(U_{\mathrm{em}}^{R_{\lambda \alpha }}+\left\langle \phi_{m}^{R_{\lambda \alpha }}|\phi_{e}\right\rangle \right)c_{\mathrm{ij}}^{\mathrm{ae}}\right] \end{array}$$

We note that the first term in addition to the last six terms in eq 9 would constitute the intermediately normalized MP2/CID AAT if one chose to use first-order amplitudes/CID coefficients and derivative first-order amplitudes/CID coefficients in place of the CISD coefficients and derivative CISD coefficients. In Stephens’s formulation of the AAT, it is assumed that the wave function is fully normalized. As such, insertion of the normalization factor *N* into eq 2 and subsequent application to eq 1 result in

10 $${\left[I_{\alpha \beta }^{\lambda }\right]}_{\mathrm{full}}=N^{2}{\left[I_{\alpha \beta }^{\lambda }\right]}_{\mathrm{int}}+N\frac{\partial N}{\partial R_{\lambda \alpha }}2\left[\sum_{\mathrm{ia}}c_{i}^{a}U_{\mathrm{ia}}^{H_{\beta }}+\sum_{\mathrm{ia}}{c_{i}^{a}}^{†}\frac{\partial c_{i}^{a}}{\partial H_{\beta }}+2\sum_{\mathrm{ia}}c_{i}^{a}\sum_{\mathrm{jb}}U_{\mathrm{jb}}^{H_{\beta }}{c_{\mathrm{ij}}^{\mathrm{ab}}}^{†}+\frac{1}{4}\sum_{\mathrm{ijab}}{c_{\mathrm{ij}}^{\mathrm{ab}}}^{†}\frac{\partial c_{\mathrm{ij}}^{\mathrm{ab}}}{\partial H_{\beta }}\right]$$

for which only the last term would contribute to a fully normalized MP2/CID formulation. In arriving at eqs 9 and 10, we have used noncanonical perturbed orbitals, which allows us to avoid numerical instabilities introduced by degeneracies (or near-degeneracies) in the orbital energies.^35^ For magnetic field perturbations, the usual relationship between the core derivative of the overlap and CPHF coefficients, *U*_*pq*_^χ^* + *U*_*qp*_^χ^ = *S*_*pq*_^χ^, becomes *U*_*pq*_^*H*_β_^* + *U*_*qp*_^*H*_β_^ = 0 since the basis functions have no dependence on magnetic fields. Orbital rotations among the occupied/occupied and virtual/virtual blocks of the CPHF coefficients result in these blocks being zero, allowing us to remove *U*_*pq*_^*H*_β_^ terms for these subspaces from the equations. Of course, this assumes that all orbitals are active; if we choose to freeze the core orbitals, we must retain any terms involving *U*_*pq*_^*H*_β_^ that contain core orbital indices in common with one or more CI coefficients. If intermediates are utilized, the terms in eqs 9 and 10 will maximally scale as *O*(*N* ^5^). For example, the last term in eq 9 can be rewritten as

11 $$\begin{array}{l} -\frac{1}{2}\sum_{\mathrm{ijab}}{c_{\mathrm{ij}}^{\mathrm{ab}}}^{†}\left[\sum_{\mathrm{me}}U_{\mathrm{bm}}^{H_{\beta }}\left(U_{\mathrm{em}}^{R_{\lambda \alpha }}+\left\langle \phi_{m}^{R_{\lambda \alpha }}|\phi_{e}\right\rangle \right)c_{\mathrm{ij}}^{\mathrm{ae}}\right]\\ =-\frac{1}{2}\sum_{\mathrm{ijab}}{c_{\mathrm{ij}}^{\mathrm{ab}}}^{†}\left[\sum_{e}X_{\mathrm{be}}c_{\mathrm{ij}}^{\mathrm{ae}}\right]=-\frac{1}{2}\sum_{\mathrm{ijab}}{c_{\mathrm{ij}}^{\mathrm{ab}}}^{†}Y_{\mathrm{ij}}^{\mathrm{ab}} \end{array}$$

where we have reduced the scaling of the equation from *O*(*N* ^6^) to *O*(*N* ^5^) . We do this by first solving for the *O*(*N* ^3^) intermediate, *X*_*be*_, which is defined as

12 $$X_{\mathrm{be}}=\sum_{m}U_{\mathrm{bm}}^{H_{\beta }}\left(U_{\mathrm{em}}^{R_{\lambda \alpha }}+\left\langle \phi_{m}^{R_{\lambda \alpha }}|\phi_{e}\right\rangle \right)$$

and then solving for the *O*(*N* ^5^) intermediate *Y*_*ij*_^*ab*^ defined as

13 $$Y_{\mathrm{ij}}^{\mathrm{ab}}=\sum_{e}X_{\mathrm{be}}c_{\mathrm{ij}}^{\mathrm{ae}}$$

The final contraction between *c*_*ij*_^*ab*†^ and *Y*_*ij*_^*ab*^ is an *O*(*N* ^4^) step. Solutions to the CI coefficient and their derivatives will still scale as *O*(*N* ^6^).

## 3 Computational Details

We have implemented the analytic-gradient method for computing CID and CISD AATs described above in the open-source Python package apyib.^38^ This package is supported by the Psi4 quantum chemistry package^39^ from which integrals and other base quantities are obtained. This choice of implementation allows for rapid development and is primarily designed to test the correctness of the approach. We compared this implementation to a finite-difference algorithm similar to that recently developed for MP2,^33^ but with derivatives of CI coefficients computed separately from the determinant overlaps as expressed in eq 5. In addition, our implementation utilizes noncanonical perturbed orbitals with the ability to invoke the frozen-core approximation, as noted above.

We have used this new implementation to examine the impact of the systematic inclusion of dynamic electron correlation by drawing comparisons of VCD rotatory strengths among the HF, MP2, CID, and CISD levels of theory computed using the aug-cc-pVDZ basis set^36,40^ for the molecular test set shown in Figure 1. All basis sets were obtained from the Basis Set Exchange.^41–43^ For the finite-difference calculations, the magnetic fields and geometric displacements were set to 10^–6^ a.u. Energies were converged to 10^–13^ a.u. for the finite-difference comparisons and to 10^–10^ a.u. or greater for the analytic evaluation of VCD quantities. Hessians and dipole derivatives (APTs) were obtained from the Gaussian quantum chemistry package.^44^ To maintain a consistent comparison between VCD rotatory strengths, we used a common-geometry/common-Hessian scheme. Core orbitals were frozen for all non-hydrogen atoms, and a narrow line width of 10^–3^ eV = 8.06573 cm^–1^ was used for the simulated VCD spectra. Our implementation does not include GIAOs and, as such, is dependent on the choice of gauge origin. To minimize these effects, we set the origin to the molecular center of mass for all calculations. We will address methods for removing the origin dependence in future work.

**Figure 1:**
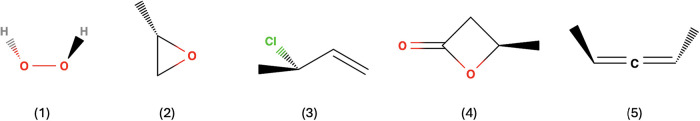
Molecular test set for comparisons of HF, MP2, CID, and CISD VCD spectra including (1) (*P*)-hydrogen peroxide, (2) (*S*)-methyloxirane, (3) (*R*)-3-chloro-1-butene, (4) (*R*)-4-methyl-2-oxetanone, and (5) (*M*)-1,3-dimethylallene.

## 4 Results and Discussion

### 4.1 Comparison between Analytic and Numerical Differentiation

To test our implementation, we compared analytic and numerical AATs for the hydrogen-molecule dimer, water, and (*P*)-hydrogen peroxide with various basis sets, and we found close agreement between the two methods. As an example, we have included the AATs computed for water using the CID level of theory in Table 1 and (*P*)-hydrogen peroxide using the CISD level of theory in Table 2, both with the 6-31G basis set.^45–47^ Errors between the implementations were on the order of 10^–9^ for (*P*)-hydrogen peroxide and 10^–8^ for (*P*)-hydrogen peroxide, which show similar agreement reported in our previous manuscript comparing the analytic approach to the finite-difference method for the MP2 case.^34^

**Table 1:** Electronic CID AATs (a.u.) for Water Computed with the 6-31G Basis Using Analytical-Gradient Methods and Finite-Difference Procedures

	analytic			finite-difference		
	*B*_*x*_	*B*_*y*_	*B*_*z*_	*B*_*x*_	*B*_*y*_	*B*_*z*_
O_1*x*_	0.0000000000	–0.1546420383	0.0000000000	–0.0000000000	–0.1546420376	–0.0000000008
O_1*y*_	0.2523864912	–0.0000000000	–0.0000000000	0.2523864918	–0.0000000001	–0.0000000002
O_1*z*_	–0.0000000000	–0.0000000000	0.0000000000	0.0000000001	–0.0000000001	0.0000000006
H_2*x*_	–0.0000000000	0.0491462089	0.0714068767	–0.0000000000	0.0491462084	0.0714068764
H_2*y*_	–0.0208338936	0.0000000000	0.0000000000	–0.0208338938	0.0000000002	0.0000000001
H_2*z*_	–0.0866781888	–0.0000000000	–0.0000000000	–0.0866781886	–0.0000000002	–0.0000000000
H_3*x*_	0.0000000000	0.0491462089	–0.0714068767	0.0000000000	0.0491462079	–0.0714068775
H_3*y*_	–0.0208338936	–0.0000000000	0.0000000000	–0.0208338942	0.0000000003	0.0000000002
H_3*z*_	0.0866781888	0.0000000000	–0.0000000000	0.0866781889	–0.0000000001	–0.0000000001

**Table 2:** Electronic CISD AATs (a.u.) for (*P*)-hydrogen Peroxide Computed with the 6-31G Basis Using Analytic-Gradient Methods and Finite-Difference Procedures

	analytic			finite-difference		
	*B*_*x*_	*B*_*y*_	*B*_*z*_	*B*_*x*_	*B*_*y*_	*B*_*z*_
H_1*x*_	–0.1898045933	–0.0222646607	0.3242355909	–0.1898045760	–0.0222646630	0.3242355780
H_1*y*_	–0.0180441509	0.0094117839	–0.0207843931	–0.0180441522	0.0094117811	–0.0207843979
H_1*z*_	–0.1840522389	0.0929224603	0.1876514299	–0.1840522373	0.0929224796	0.1876514376
H_2*x*_	–0.1898045933	–0.0222646607	–0.3242355909	–0.1898045765	–0.0222646557	–0.3242355864
H_2*y*_	–0.0180441509	0.0094117839	0.0207843931	–0.0180441571	0.0094117735	0.0207843885
H_2*z*_	0.1840522389	–0.0929224603	0.1876514299	0.1840522317	–0.0929224552	0.1876514226
O_3*x*_	–0.0451458652	0.0794663609	1.1847772282	–0.0451458859	0.0794663660	1.1847772322
O_3*y*_	–0.1508788119	–0.0042901597	0.0160726896	–0.1508787987	–0.0042901616	0.0160726887
O_3*z*_	–1.1567011160	–0.0219982861	0.0422548261	–1.1567011357	–0.0219982849	0.0422548271
O_4*x*_	–0.0451458652	0.0794663609	–1.1847772282	–0.0451458794	0.0794663859	–1.1847772241
O_4*y*_	–0.1508788119	–0.0042901597	–0.0160726896	–0.1508788143	–0.0042901346	–0.0160726874
O_4*z*_	1.1567011160	0.0219982861	0.0422548261	1.1567011203	0.0219982969	0.0422548247

### 4.2 VCD Spectrum of (*P*)-hydrogen Peroxide

Using the recently developed analytic derivative formulation for computing MP2 AATs and the CID/CISD AAT approaches derived here, we compare VCD spectra and the requisite rotatory strengths for (*P*)-hydrogen peroxide using the HF, MP2, CID, and CISD levels of theory. (*P*)-hydrogen peroxide is included in our molecular test set due to its small size and axial chirality. We find rather distinct differences between the four spectra, even for a small molecule such as (*P*)-hydrogen peroxide. The vibrational frequencies and corresponding rotatory strengths are presented in Table 3 and the VCD spectrum is in Figure 2.

**Figure 2:**
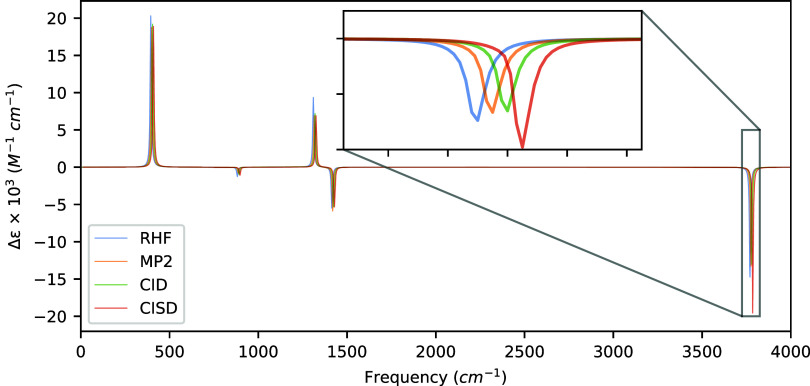
VCD spectra of (*P*)-hydrogen peroxide computed at the HF, MP2, and CISD levels of theory with aug-cc-pVDZ using a common optimized geometry and Hessian obtained at the MP2/aug-cc-pVDZ level of theory. Artificial shifts of 5 cm^–1^, 10 cm^–1^, and 15 cm^–1^ for MP2, CID, and CISD, respectively, have been introduced into the spectrum to distinguish the peaks more easily.

**Table 3:** VCD Rotatory Strengths of (*P*)-hydrogen Peroxide Computed at the HF, MP2, CID, and CISD Levels of Theory with aug-cc-pVDZ Using a Common Optimized Geometry and Hessian Obtained at the MP2/aug-cc-pVDZ Level

	rotatory strength (10^–44^ esu^2^ cm^2^)			
frequency (cm^–1^)	HF	MP2	CID	CISD
3768.80	–17.909	–17.970	–17.416	–29.793
3767.46	7.091	8.738	8.312	17.149
1414.00	–11.330	–12.111	–10.977	–10.995
1309.92	20.802	15.368	15.984	15.344
881.29	–4.304	–2.759	–3.454	–3.688
394.22	150.336	138.916	142.114	139.937

The most notable differences between the HF and MP2 levels of theory occur at 394.22 cm^–1^ and 1309.92 cm^–1^, which are related to hydrogen bending motions perpendicular and parallel to the O–O bond axis, respectively. For these vibrational modes, the MP2 rotatory strengths are predicted to be ∼11 × 10^–44^ esu^2^ cm^2^ and ∼5 × 10^–44^ esu^2^ cm^2^ smaller than those from the uncorrelated wave function. CID predicts similar rotational strengths; however, optimization of the CI coefficients shifts the rotational strengths for the 394.22 cm^–1^ mode back in the direction of that produced by the HF wave function. Likewise, the CISD rotatory strengths for these modes are similar to those predicted by the MP2 method. Interestingly, we note substantial differences in rotatory strengths between the MP2 and CID methods versus CISD for the nearly degenerate in-phase and out-of-phase hydrogen bond stretches at 3767.46 cm^–1^ and 3768.80 cm^–1^, respectively. Both of these modes exhibit rotatory strengths of almost an order of magnitude more intense for the CISD method over that of MP2 and CID—a direct consequence of including singly excited determinants. The differences between these nearly degenerate modes are not as noticeable in the spectra due to the fact that the peaks have opposite signs leading to some cancellation between them, depending on the spectral resolution as shown in the inset of Figure 2.

### 4.3 VCD Spectrum of (*S*)-methyloxirane

We include (*S*)-methyloxirane in our molecular test set due to its historical significance as a troublesome test case for optical properties, its conformational rigidity, and unique bonding structure as a member of the oxirane family of molecules. In Table 4 and Figure 3, we present the rotatory strengths and VCD spectra of (*S*)-methyloxirane computed at the HF, MP2, CID, and CISD levels of theory. Two vibrational modes are noteworthy: 1183.14 cm^–1^, which is a rocking motion of all of the hydrogen atoms, and 1140.36 cm^–1^, which is the mode associated with a bending motion of the hydrogen atoms on the CH_2_ carbon atom parallel to the plane of the epoxide ring. The former exhibits a sign discrepancy among HF, CID, and CISD versus MP2, while the latter exhibits a similar discrepancy between HF and the correlated methods, as shown in the inset of Figure 3. Both modes produce rotatory strengths less than 1 × 10^–44^ esu^2^ cm^2^ for each of the methods tested which, given their relative weakness, makes the sign discrepancies relatively unsurprising. Interestingly, the modes most affected by the inclusion of singly excited determinants into the wave function are hydrogen stretching motions of 3158.52 cm^–1^ and 3175.86 cm^–1^. In these modes, we note a shift in rotatory strength of about 1–1.5 × 10^–44^ esu^2^ cm^2^ between the CID and CISD methods, providing evidence for a small contribution to the rotatory strengths and VCD spectra from inclusion of singly excited determinants into the wave function for (*S*)-methyloxirane.

**Figure 3:**
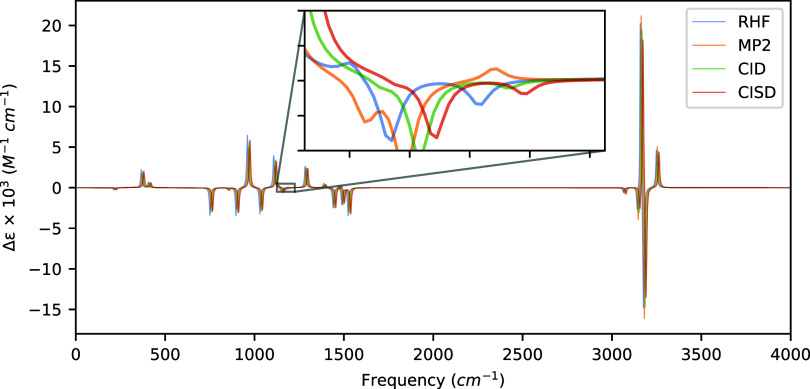
VCD spectra of (*S*)-methyloxirane computed at the HF, MP2, and CISD levels of theory with aug-cc-pVDZ using a common optimized geometry and Hessian obtained at the MP2/aug-cc-pVDZ level of theory. Artificial shifts of 5 cm^–1^, 10 cm^–1^, and 15 cm^–1^ for MP2, CID, and CISD, respectively, have been introduced into the spectrum to distinguish the peaks more easily.

**Table 4:** VCD Rotatory Strengths of (*S*)-methyloxirane Computed at the HF, MP2, CID, and CISD Levels of Theory with aug-cc-pVDZ Using a Common Optimized Geometry and a Hessian Obtained at the MP2/aug-cc-pVDZ Level

	rotatory strength (10^–44^ esu^2^ cm^2^)			
frequency (cm^–1^)	HF	MP2	CID	CISD
3248.50	4.202	4.640	4.090	4.010
3175.86	–14.496	–15.784	–14.379	–13.225
3158.52	23.028	24.315	22.291	20.602
3154.34	–5.751	–6.226	–5.666	–4.926
3141.40	–3.124	–4.287	–3.421	–2.923
3063.53	–0.566	–0.674	–0.624	–0.765
1523.43	–6.659	–5.626	–6.184	–6.236
1488.22	–4.004	–4.054	–3.893	–3.642
1471.44	0.770	0.880	0.798	0.755
1439.22	–5.082	–5.262	–5.109	–5.057
1384.01	0.750	1.153	0.967	0.810
1283.08	6.265	5.715	5.746	5.583
1183.14	–0.453	0.226	–0.137	–0.250
1153.29	–1.208	–1.688	–1.472	–1.113
1140.36	0.279	–0.724	–0.130	–0.103
1106.88	10.859	7.497	9.396	9.032
1029.37	–9.447	–8.055	–8.583	–7.938
959.19	20.484	15.893	18.032	18.510
896.28	–11.327	–8.763	–9.988	–10.073
843.85	–0.107	–0.815	–0.528	–1.170
750.52	–13.478	–10.745	–11.862	–11.393
405.53	4.797	4.325	4.445	4.187
365.86	17.634	14.357	15.986	16.124
212.36	–3.652	–3.346	–3.427	–3.497

### 4.4 VCD Spectrum of (*R*)-3-chloro-1-butene

The rotatory strengths and VCD spectra of (*R*)-3-chloro-1-butene are reported in Table 5 and Figure 4, respectively. (*R*)-3-chloro-1-butene is unique among our test molecules because it includes both double bond character and a halogen atom. Of the 30 vibrational modes associated with (*R*)-3-chloro-1-butene, only two modes exhibit sign changes among the four levels of theory examined here. In the first mode at 1000.49 cm^–1^, a hydrogen bending vibration, we note a sign change of the MP2 method relative to all others, as illustrated by the inset of Figure 4. Likewise, the vibrational mode at 3195.37 cm^–1^, a C–H stretching vibration of hydrogens associated with the C=C double bond, deviates in its sign at the HF level relative to CID, MP2, and CISD. However, both of these vibrations exhibit very weak rotatory strengths, similarly to the case of (*S*)-methyloxirane above. Additionally, two modes at 1254.14 cm^–1^ and 885.18 cm^–1^, the former a hydrogen bending motion on the Cl–C–H bond angle and the latter a more complex mode involving combinations of C–H bending and C–C stretching motions, are the most affected by the inclusion of singly excited determinants into the wave function, though the shift is small—less than 1 × 10^–44^ esu^2^ cm^2^ relative to the CID method, indicating that singly excited determinants do not significantly contribute to the rotatory strengths of (*R*)-3-chloro-1-butene.

**Figure 4:**
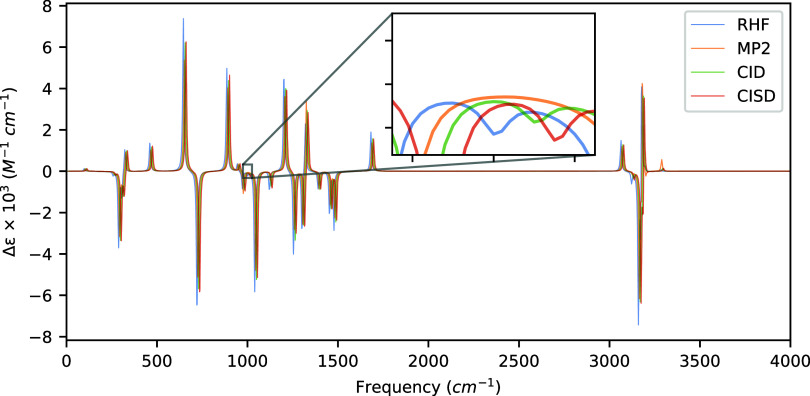
VCD spectra of (*R*)-3-chloro-1-butene computed at the HF, MP2, and CISD levels of theory with aug-cc-pVDZ using a common optimized geometry and Hessian obtained at the MP2/aug-cc-pVDZ level of theory. Artificial shifts of 5 cm^–1^, 10 cm^–1^, and 15 cm^–1^ for MP2, CID, and CISD, respectively, have been introduced into the spectrum to distinguish the peaks more easily.

**Table 5:** VCD Rotatory Strengths of (*R*)-3-chloro-1-butene Computed at the HF, MP2, CID, and CISD Levels of Theory with aug-cc-pVDZ Using a Common Optimized Geometry and a Hessian Obtained at the MP2/aug-cc-pVDZ Level

	rotatory strength (10^–44^ esu^2^ cm^2^)			
frequency (cm^–1^)	HF	MP2	CID	CISD
3283.33	0.064	0.514	0.127	0.132
3195.37	0.194	–0.292	–0.044	–0.092
3175.65	13.563	11.129	10.946	10.663
3173.97	–12.168	–8.545	–9.140	–8.890
3159.73	–6.780	–5.733	–5.817	–5.880
3122.02	–0.507	–0.127	–0.273	–0.341
3063.13	1.486	1.204	1.284	1.266
1681.99	3.274	2.372	2.716	2.695
1481.56	1.397	0.497	0.892	0.671
1477.73	–6.342	–4.651	–5.263	–4.956
1452.78	–4.101	–3.285	–3.624	–3.674
1389.58	–1.786	–1.776	–1.850	–1.790
1320.00	5.363	7.708	6.757	6.552
1300.95	–6.692	–6.321	–6.356	–6.494
1254.14	–9.338	–6.557	–7.771	–6.986
1201.01	11.477	9.319	10.305	10.100
1120.95	–2.376	–1.171	–1.968	–2.198
1040.08	–16.312	–13.395	–14.644	–14.381
1000.49	–0.484	0.005	–0.331	–0.578
971.74	–2.537	–3.236	–2.705	–2.864
943.63	1.132	1.091	1.048	1.154
885.18	17.036	13.795	15.014	15.902
720.96	–27.735	–21.947	–24.329	–24.977
645.30	34.303	24.976	28.860	29.071
460.50	8.695	6.600	7.447	7.795
321.73	10.527	9.282	9.946	9.977
304.83	–11.310	–9.569	–10.611	–10.526
287.72	–37.090	–31.644	–33.486	–33.682
257.56	–2.016	–1.216	–1.678	–1.718
97.78	3.750	3.321	3.729	3.709

### 4.5 VCD Spectrum of (*R*)-4-methyl-2-oxetanone

Table 6 and Figure 5 present the rotatory strengths and VCD spectra of (*R*)-4-methyl-2-oxetanone, respectively. Our choice to include (*R*)-4-methyl-2-oxetanone in our molecular test set is primarily due to its rigid structure and unique combination of functional groups, including a ketone attached to a four-membered ring involving an ether linkage. We find two interesting peaks with sign discrepancies between the methods. The most significant sign discrepancy of our test set, a carbonyl stretch at 1859.68 cm^–1^, is predicted by the MP2 method to have a rotational strength of +3.611 × 10^–44^ esu^2^ cm^2^ while HF, CID, and CISD predicted negative values of −4.749 × 10^–44^ esu^2^ cm^2^, −1.560 × 10^–44^ esu^2^ cm^2^, and −2.856 × 10^–44^ esu^2^ cm^2^, respectively, as shown in the inset of Figure 5. Likewise, the inclusion of singly excited determinants shifts the rotational strength by about −1.3 × 10^–44^ esu^2^ cm^2^ from the CID to CISD methods—one of the more significant deviations observed thus far. The second mode, a ring breathing motion at 704.03 cm^–1^, is predicted by the HF method to have a positive sign, while the MP2, CID, and CISD methods predict negative signs, though this mode is much weaker. In addition to the mode at 1859.68 cm^–1^, we observe two other modes affected by the inclusion of singly excited determinants at 1297.72 cm^–1^ and 1035.89 cm^–1^, the former a C–H bending motion and the latter a bending motion involving both the C=O double bond and the methyl group. The shifts in the rotatory strengths between the CID and CISD methods are between 1 and 1.5 × 10^–44^ esu^2^ cm^2^ for both modes, indicating, again, only a slight effect of the inclusion of singly excited determinants into the wave function for (*R*)-4-methyl-2-oxetanone.

**Figure 5:**
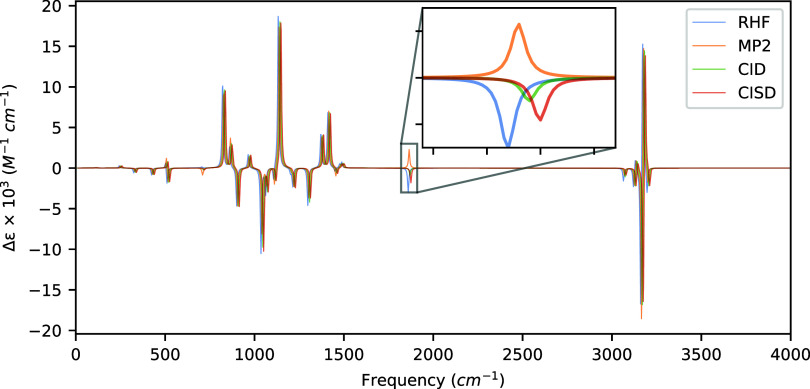
VCD spectra of (*M*)-1,3-dimethylallene computed at the HF, MP2, and CISD levels of theory with aug-cc-pVDZ using a common optimized geometry and Hessian obtained at the MP2/aug-cc-pVDZ level of theory. Artificial shifts of 5 cm^–1^, 10 cm^–1^, and 15 cm^–1^ for MP2, CID, and CISD, respectively, have been introduced into the spectrum to distinguish the peaks more easily.

**Table 6:** VCD Rotatory Strengths of (*R*)-4-methyl-2-oxetanone Computed at the HF, MP2, CID, and CISD Levels of Theory with aug-cc-pVDZ Using a Common Optimized Geometry and Hessian Obtained at the MP2/aug-cc-pVDZ Level

	rotatory strength (10^–44^ esu^2^ cm^2^)			
frequency (cm^–1^)	HF	MP2	CID	CISD
3195.99	–2.934	–1.788	–2.173	–2.146
3171.17	16.723	16.350	15.917	15.277
3160.57	–17.647	–19.338	–17.636	–17.197
3130.64	1.347	1.327	1.228	0.960
3119.72	–2.239	–2.057	–2.129	–2.073
3063.98	–1.495	–0.834	–0.994	–0.806
1859.68	–4.749	3.611	–1.560	–2.856
1487.08	1.422	0.761	1.099	0.910
1476.00	0.853	0.760	0.877	0.946
1449.43	–0.990	–2.184	–1.410	–1.575
1409.39	14.335	14.727	14.300	14.046
1370.99	9.175	8.432	8.800	9.055
1297.72	–10.422	–8.312	–9.500	–8.333
1213.11	–5.836	–5.691	–5.739	–6.115
1201.03	–1.503	–0.950	–1.263	–0.824
1132.78	50.039	46.530	48.258	47.860
1106.25	–4.509	–6.141	–4.948	–5.026
1061.42	–8.951	–5.813	–7.699	–7.669
1035.89	–29.483	–22.713	–27.232	–28.667
964.31	5.144	4.310	4.761	4.960
899.66	–15.386	–15.445	–15.453	–15.613
860.03	8.614	12.292	9.961	9.435
821.12	37.429	33.642	35.619	35.198
704.03	0.565	–3.580	–1.029	–1.022
509.75	–11.831	–11.399	–11.395	–10.904
501.83	5.994	9.434	6.999	6.756
424.19	–5.861	–5.242	–5.458	–5.474
323.72	–5.418	–3.786	–4.710	–4.903
242.97	3.460	3.056	3.240	3.277
103.73	2.033	2.081	2.028	2.018

### 4.6 VCD Spectrum of (*M*)-1,3-dimethylallene

In Table 7 and Figure 6, we present the rotatory strengths and VCD spectra for (*M*)-1,3-dimethylallene. (*M*)-1,3-dimethylallene is also a good addition to our test set because of its unique bonding structure involving adjacent double bonds and a helical molecular orbital structure. In contrast to the other molecules in our test set, the inclusion of singly excited determinants plays a significant role in the spectroscopic simulation of the VCD for (*M*)-1,3-dimethylallene. Most notable is the vibrational mode at 693.06 cm^–1^—a mode associated with a bending motion of the hydrogen atoms on the allene moiety. Interestingly, HF and CID methods predict this mode to exhibit a negative rotatory strength, while the MP2 and CISD levels of theory predicted a positive value, though all four methods predict small values, as illustrated by the inset of Figure 6.

**Figure 6:**
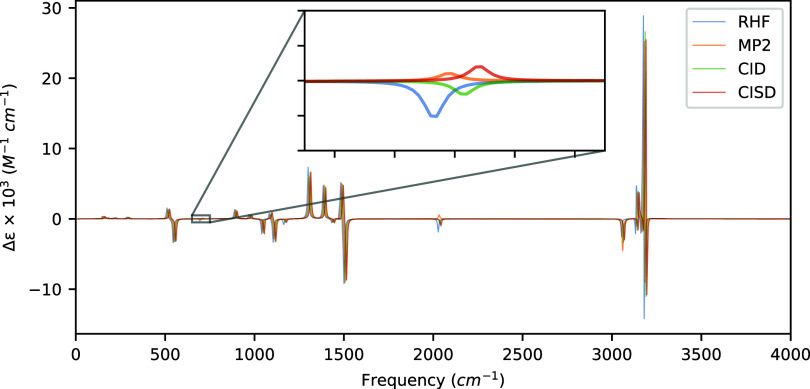
VCD spectra of (*M*)-1,3-dimethylallene computed at the HF, MP2, and CISD levels of theory with aug-cc-pVDZ using a common optimized geometry and Hessian obtained at the MP2/aug-cc-pVDZ level of theory. Artificial shifts of 5 cm^–1^, 10 cm^–1^, and 15 cm^–1^ for MP2, CID, and CISD, respectively, have been introduced into the spectrum to distinguish the peaks more easily.

**Table 7:** VCD Rotatory Strengths of (*M*)-1,3-dimethylallene Computed at the HF, MP2, CID, and CISD Levels of Theory with aug-cc-pVDZ Using a Common Optimized Geometry and a Hessian Obtained at the MP2/aug-cc-pVDZ Level

	rotatory strength (10^–44^ esu^2^ cm^2^)			
frequency (cm^–1^)	HF	MP2	CID	CISD
3177.99	–123.004	–95.171	–105.805	–102.292
3176.99	133.216	105.986	116.353	112.238
3163.83	6.123	1.049	3.132	2.963
3163.03	–9.216	–4.356	–6.139	–5.800
3134.81	184.936	126.346	150.405	143.942
3134.69	–183.138	–124.610	–148.863	–142.436
3056.10	8.233	7.031	7.259	6.440
3055.54	–10.203	–11.287	–10.229	–9.082
2027.70	–2.672	0.813	–1.192	–1.435
1499.71	–18.434	–18.252	–17.764	–17.398
1481.80	10.876	10.617	10.310	10.058
1471.33	6.098	6.054	5.855	5.355
1470.28	–5.718	–5.742	–5.498	–4.987
1431.97	–1.201	–1.019	–1.175	–1.214
1385.40	–7.362	–9.321	–7.999	–7.456
1384.16	16.421	18.446	16.876	15.914
1299.51	16.623	13.499	14.832	15.114
1163.04	–2.035	–0.137	–1.237	–1.273
1103.82	–8.886	–7.539	–8.344	–8.670
1083.04	2.535	1.951	2.367	2.933
1043.14	4.624	3.466	3.939	4.202
1040.68	–9.120	–7.653	–8.233	–8.799
968.74	1.970	1.551	1.716	1.774
887.61	4.281	4.279	4.067	3.582
824.20	0.195	0.096	0.127	0.145
693.05	–1.124	0.231	–0.416	0.446
544.89	–19.123	–18.133	–18.168	–17.939
509.78	9.067	7.686	8.171	8.120
284.38	2.411	2.322	2.316	2.083
210.94	1.915	1.617	2.099	1.853
167.53	2.366	1.940	2.069	2.113
148.40	7.089	6.985	6.617	6.764
129.93	–1.014	–1.015	–0.958	–1.025

The intense pairs of modes at 3134.69/3134.81 cm^–1^ and at 3176.99/3177.99 cm^–1^ exhibit the largest absolute shifts in rotatory strengths between CID and CISD, as well as the largest shifts due to dynamic electron correlation effects. The rotatory strengths for these modes, which correspond to in-phase/out-of-phase C–H stretches (the former pair on the cumulene chain and the latter on the methyl groups), occur in opposite-sign pairs and thus exhibit significant cancellation in the VCD spectra. The inclusion of single excitations between CID and CISD reduces the magnitude of the rotatory strengths of the methyl C–H stretches by 3–4 × 10^–44^ esu^2^ cm^2^ and those of the cumulenic C–H stretches by 6–7 × 10^–44^ esu^2^ cm^2^. Additionally, the shift in the rotatory strengths between HF and the correlated methods is even larger: for the methyl C–H stretches, the intensities are reduced in magnitude by ca. 28 × 10^–44^ esu^2^ cm^2^, while that for the cumulenic C–H stretched by almost 59 × 10^–44^ esu^2^ cm^2^ between HF and MP2. We observe an additional sign discrepancy for the weak mode at 2027.70 cm^–1^ that corresponds to vibration of the two C=C double bonds. For this mode, HF, CID, and CISD predict the mode to have a negative sign between −2.672 × 10^–44^ esu^2^ cm^2^ and −1.192 × 10^–44^ esu^2^ cm^2^ while MP2 predicts a positive value of 0.813 × 10^–44^ esu^2^ cm^2^.

## 5 Conclusions

Based on a second-quantized formalism for derivatives of Slater determinants, we have obtained expressions for the first time for CID- and CISD-level AATs. Our formulation uses noncanonical perturbed orbitals, allows for frozen-core orbitals, and scales as $O\left(N^{5}\right)$ . We have validated the analytic derivative implementation against corresponding numerical derivatives and observed excellent agreement. Additionally, we have compared HF, MP2, CID, and CISD rotatory strengths and VCD spectra for (*P*)-hydrogen peroxide, (*S*)-methyloxirane, (*R*)-3-chloro-1-butene, (*R*)-4-methyl-2-oxetanone, and (*M*)-1,3-dimethylallene using the aug-cc-pVDZ basis. For four of the five test molecules, we observed sign differences between the HF and MP2 methods relative to CID and CISD. Similarly, the inclusion of singly excited determinants into the wave function for (*P*)-hydrogen peroxide and (*M*)-1,3-dimethylallene significantly affects the rotatory strengths of peaks in the high-frequency region, although near degeneracy of the corresponding modes suppresses these effects somewhat in the resulting spectra. Additionally, we observed a sign change between the CID and CISD for (*M*)-1,3-dimethylallene for a weak mode in the mid-IR region.

Curiously, we observed that the molecules most affected by the inclusion of singly excited determinants into the wave function are those which exhibit axial chirality ((*P*)-hydrogen peroxide and (*M*)-1,3-dimethylallene) rather than point chirality ((*S*)-methyloxirane, (*R*)-3-chloro-1-butene, and (*R*)-4-methyl-2-oxetanone), though determining whether this is a general phenomenon will require additional investigation. We will consider this topic in future work, as well as the important issue of gauge-origin dependence of the rotatory strengths.

## Supplementary Material



## Notes

Atomic coordinates of all test molecules (PDF)
