# Supplementary material for: Analytic Computation of Vibrational Circular Dichroism Spectra Using Configuration Interaction Methods
Source: J Phys Chem A. 2026 Jan 15;130(4):973–83. doi: 10.1021/acs.jpca.5c07287 (PMC12862817; doi:10.1021/acs.jpca.5c07287)
Supplement: Supplementary file 1 [file jp5c07287_si_001.pdf]

Analytic Computation of Vibrational Circular Dichroism Spectra Using Configuration Interaction Methods

Brendan M. Shumberger and T. Daniel Crawford*

Department of Chemistry, Virginia Tech, Blacksburg, Virginia, U.S.A.

E-mail: crawdad@vt.edu

Table S1: Water MP2/aug-cc-pVDZ optimized geometry (a.u.)

Atom Type	Atom Number	X	Y	Z
O	1	0.000000000	0.000000000	0.225076620
H	2	-0.000000000	1.437143920	-0.900306479
H	3	-0.000000000	-1.437143920	-0.900306479

Table S2: (P)-hydrogen peroxide MP2/aug-cc-pVDZ optimized geometry (a.u.)

Atom Type	Atom Number	X	Y	Z
H	1	1.510969300	1.680961660	0.890298686
H	2	-1.510969300	-1.680961660	0.890298686
O	3	0.000000000	1.389172190	-0.111287336
O	4	-0.000000000	-1.389172190	-0.111287336

Table S3: (S)-methyloxirane MP2/aug-cc-pVDZ optimized geometry (a.u.)

Atom Type	Atom Number	X	Y	Z
O	1	-1.574608840	-1.517705290	-0.449954037
C	2	0.295002686	-0.053243893	0.939472664
C	3	-1.962316090	1.190664630	-0.123225333
C	4	2.853831210	0.180280598	-0.288047312
H	5	0.280228106	-0.458244226	2.972442350
H	6	-3.546091340	1.685780610	1.109440330
H	7	-1.768991360	2.295411350	-1.861636110
H	8	3.945428090	1.714308110	0.603302097
H	9	3.925491970	-1.591016280	-0.077818482
H	10	2.641698420	0.589194789	-2.315297990

Table S4: (R)-3-chloro-1-butene MP2/aug-cc-pVDZ optimized geometry (a.u.)

Atom Type	Atom Number	X	Y	Z
C	1	-0.855826530	3.296432300	-0.092027451
C	2	-0.026259542	0.629182704	0.621489399
C	3	2.249379540	-0.237748639	-0.824178725
C	4	4.436788270	-0.916026720	0.300928066
H	5	4.618880560	-0.922755075	2.360744410
H	6	-2.573558560	3.837399370	0.945425920
H	7	6.082277130	-1.481948070	-0.809727310
H	8	2.075683760	-0.255559516	-2.889583350
H	9	0.309996895	0.466387413	2.667360750
H	10	-1.246306320	3.407006530	-2.132349160
H	11	0.671440419	4.634135090	0.373307953
Cl	12	-2.633112020	-1.547982560	-0.032496879

Table S5: (R)-4-methyl-2-oxetanone MP2/aug-cc-pVDZ optimized geometry (a.u.)

Atom Type	Atom Number	X	Y	Z
C	1	0.191035681	2.195806280	0.180236659
C	2	2.030372590	-0.019603472	-0.070473015
O	3	0.260676582	-1.834238420	0.623618541
C	4	-1.736291380	0.140561703	0.916897913
C	5	-3.919152140	-0.334914775	-0.865164592
H	6	-4.851020560	-2.137809860	-0.415360371
H	7	-5.319051580	1.197117300	-0.688884819
H	8	-3.239980490	-0.405088770	-2.830553750
H	9	-2.321988990	0.181703291	2.910411020
O	10	4.213957670	-0.348125745	-0.623747602
H	11	0.722047100	3.546504910	1.663057130
H	12	-0.182867911	3.185388020	-1.606618520

Table S6: (M)-1,3-dimethylallene MP2/aug-cc-pVDZ optimized geometry (a.u.)

Atom Type	Atom Number	X	Y	Z
C	1	-4.418643010	0.887202760	0.363394103
C	2	-2.393139590	-0.793484683	-0.755355269
C	3	0.000004942	-0.807718874	-0.000008225
C	4	2.393150120	-0.793508776	0.755346339
H	5	2.949395250	-2.080782050	2.283420730
C	6	4.418633660	0.887216063	-0.363378587
H	7	5.250657870	2.110222840	1.104005400
H	8	5.959952570	-0.259436496	-1.170023830
H	9	3.632349900	2.090884410	-1.863823710
H	10	-2.949389740	-2.080714480	-2.283465980
H	11	-5.250636970	2.110257670	-1.103965900
H	12	-5.959972930	-0.259487886	1.169963600
H	13	-3.632392730	2.090817060	1.863899520
